# Are there associations between hip geometry and bone quality? An analysis on 3074 adults from a general population

**DOI:** 10.1007/s00402-023-05031-5

**Published:** 2023-08-30

**Authors:** Cornelius Sebastian Fischer, Till Ittermann, Anke Hannemann, Carsten Oliver Schmidt, Moritz Mederake, Daniel Schüll, Tina Histing, Jörn Lange, Lyubomir Haralambiev

**Affiliations:** 1 Department of Traumatology and Reconstructive Surgery, BG Unfallklinik Tübingen, Eberhard Karls University Tübingen, Schnarrenbergstraße 95, 72076 Tübigen, Germany; 2 Institute for Community Medicine, University Medicine Greifswald, Greifswald, Germany; 3 Center for Orthopaedics, Trauma Surgery and Rehabilitation Medicine, University Medicine Greifswald, Greifswald, Germany; 4 Department of Trauma and Orthopaedic Surgery, BG Klinikum Unfallkrankenhaus Berlin, Berlin, Germany; 5 Institute of Clinical Chemistry and Laboratory Medicine, University Medicine Greifswald, Greifswald, Germany; 6 DZHK (German Centre for Cardiovascular Research), Partner Site Greifswald, University Medicine, Greifswald, Germany

**Keywords:** Hip geometry, Bone quality, Fracture risk, Quantitative ultrasound, Center-edge angle, Neck-shaft angle, Alpha angle

## Abstract

**Introduction:**

Patients with reduced bone mineral density and altered hip geometry are susceptible for hip pathologies. Knowledge on associations between bone properties and hip geometric parameters might facilitate identification of patients at risk for hip pathologies. The aim of the present study was to identify associations of bone properties assessed by quantitative ultrasound (QUS) at the heel and hip geometric parameters like center-edge angle (CE), neck-shaft angle (NSA) and alpha angle.

**Materials and methods:**

Hip geometric parameters (CE, NSA and alpha angle) of 3074 participants from the population-based Study of Health in Pomerania were assessed on magnetic resonance imaging. QUS was performed on both calcanei providing broadband ultrasound attenuation (BUA), speed of sound (SOS) and stiffness-index. Based on the stiffness-index the individual osteoporotic fracture risk (low, moderate or high) was determined. Associations between QUS-based and hip geometric parameters were calculated in linear regression models adjusted for age, sex, body height and weight. Interactions of QUS markers with age and sex on hip geometric parameters were tested.

**Results:**

Significant inverse associations between BUA (β = − 0.068), SOS (β = − 0.024) as well as stiffness-index (β = − 0.056) and CE were present, while fracture risk was positively associated with CE (β for high = 1.28 and moderate = 2.54 vs. low fracture risk). Interactions between BUA and sex as well as between SOS and age were detected in the models for CE. Furthermore, there was an inverse relation between fracture risk and NSA that was restricted to the moderate risk (β for moderate vs. low fracture risk = − 0.60). There were no significant associations between QUS parameters and alpha angle.

**Conclusions:**

In the general population, several associations between QUS-based bone properties or fracture risk and hip geometry are present. Less dysplastic hips had a lower stiffness-index and a higher fracture risk, whereas more valgus hips had a lower fracture risk.

## Introduction

Hip pathologies are common among people over 50 years and are associated with high economic costs. In Germany, osteoarthritis, with a prevalence of 6.2% in 2014 (health insurance “BARMER” claims data) [1] and hip fractures, with an incidence of over 100 per 100,000 inhabitants in 2019 for both pertrochanteric and femoral neck fractures, are increasing with age [2]. Therefore, these pathologies constitute important health issues with regard to Germany’s aging society [3–5]. Furthermore, osteoarthritis and hip fractures often require surgical therapy. Knowing the risk factors and their interactions as well as having suitable screening tools is mandatory for prevention of these diseases.

Previously, geometric parameters of the proximal femur on radiographs and their relation with osteoarthritis were studied [6–11]. The focus of these studies laid on femoroacetabular impingement (FAI), hip dysplasia and the femoral neck-shaft angle (NSA). FAI was found to be a major cause of hip osteoarthritis irrespectively of whether arising from Cam impingement, Pincer impingent, or both [6, 7]. Another important risk factor for osteoarthritis is hip dysplasia. Studies have shown that patients with center-edge angle (CE) ≤ 20° have a higher risk of developing hip osteoarthritis than patients without hip dysplasia (CE > 20°) [8]. Increased bone mineral density (BMD) also seems to be associated with osteoarthritis [12, 13]. Additionally, the CE as well as BMD may have prognostic value for subchondral insufficiency fractures of the femoral head [14].

Results regarding the relationship between NSA and hip osteoarthritis are contradictory. While some studies found coxa valga in an early stage of osteoarthritis or even determined it as a geometrical risk factor [9], others described opposing results [10, 11]. Low BMD is a major risk factor for proximal femur fractures [15, 16]. However, the combination of BMD and geometric parameters seems to improve the risk prediction for these fractures [17–19].

According to the World Health Organization, osteoporosis is diagnosed as decreased BMD evaluated by dual X-ray absorptiometry (DXA) at the spine or the hip [20]. Another approach to assess bone properties is via quantitative ultrasound (QUS). In contrast to DXA measurements, QUS is simple, quick, inexpensive, and free from ionizing radiation. It measures the speed of sound (SOS) and the broadband ultrasound attenuation (BUA) of an ultrasound signal passing through bone. From these two parameters, a combined score called stiffness-index can be calculated [21]. The stiffness-index can be used to estimate the individual osteoporotic fracture risk. QUS measurements can be performed at different skeletal sites. Because of its easy accessibility and its bone structure, the calcaneus is the most studied site [22]. Although QUS measurements are not directly comparable to DXA, there are several studies proving QUS to be a reliable predictor of fracture risk [23, 24] and useful for early diagnosis of osteoporosis [25]

Overall, patients with reduced bone quality and altered hip geometry represent a vulnerable collective for pathologies of the proximal femur and the hip. Therefore, knowledge on the associations of bone quality and hip geometry might be valuable. Thus, the aim of our study was to identify associations between QUS-based bone properties and hip geometry parameters derived from magnetic resonance imaging (MRI) including CE, NSA and alpha angle in a population-based setting.

## Methods

### Design and sample

The Study of Health in Pomerania (SHIP) is an ongoing population-based project. It consists of two independent cohorts, SHIP-START and SHIP-TREND. To ensure a general population cohort, participants were randomly recruited from official resident registry office files and stratified by sex, age and city of residence. The samples for both cohorts were drawn from a defined region in Northeastern Germany. In the SHIP-START cohort 4308 individuals participated in baseline assessments between 1997 and 2001 (SHIP-START-0, response of 68.8%). The five- and ten-year follow-up examinations took place between 2002 and 2006 (SHIP-START-1; n = 3300) and between 2008 and 2012 (SHIP-START-2; n = 2333). The sample for the second cohort (SHIP-TREND) was drawn in 2008 with the same stratification variables. In the baseline assessments, SHIP-TREND-0, 4420 individuals were examined (response 50.1%) [26].

For the present study, all participants from SHIP-START-2 and SHIP-TREND-0 with a completed hip MRI protocol were included. Overall, 3317 out of 6753 volunteers participated in the MRI examination. Drop-outs were caused, for instance, by claustrophobia, metal implants or personal reasons. Of 3317 participants, 34 interrupted their examination due to acute problems. Furthermore, 57 of 3283 completed pelvic MRIs had to be excluded because of missing data (36), total hip arthroplasty (18), extreme deformity (2) or suboptimal quality (1). In total, bilateral measurements were possible on 3226 MRIs.

**Fig. 1:**
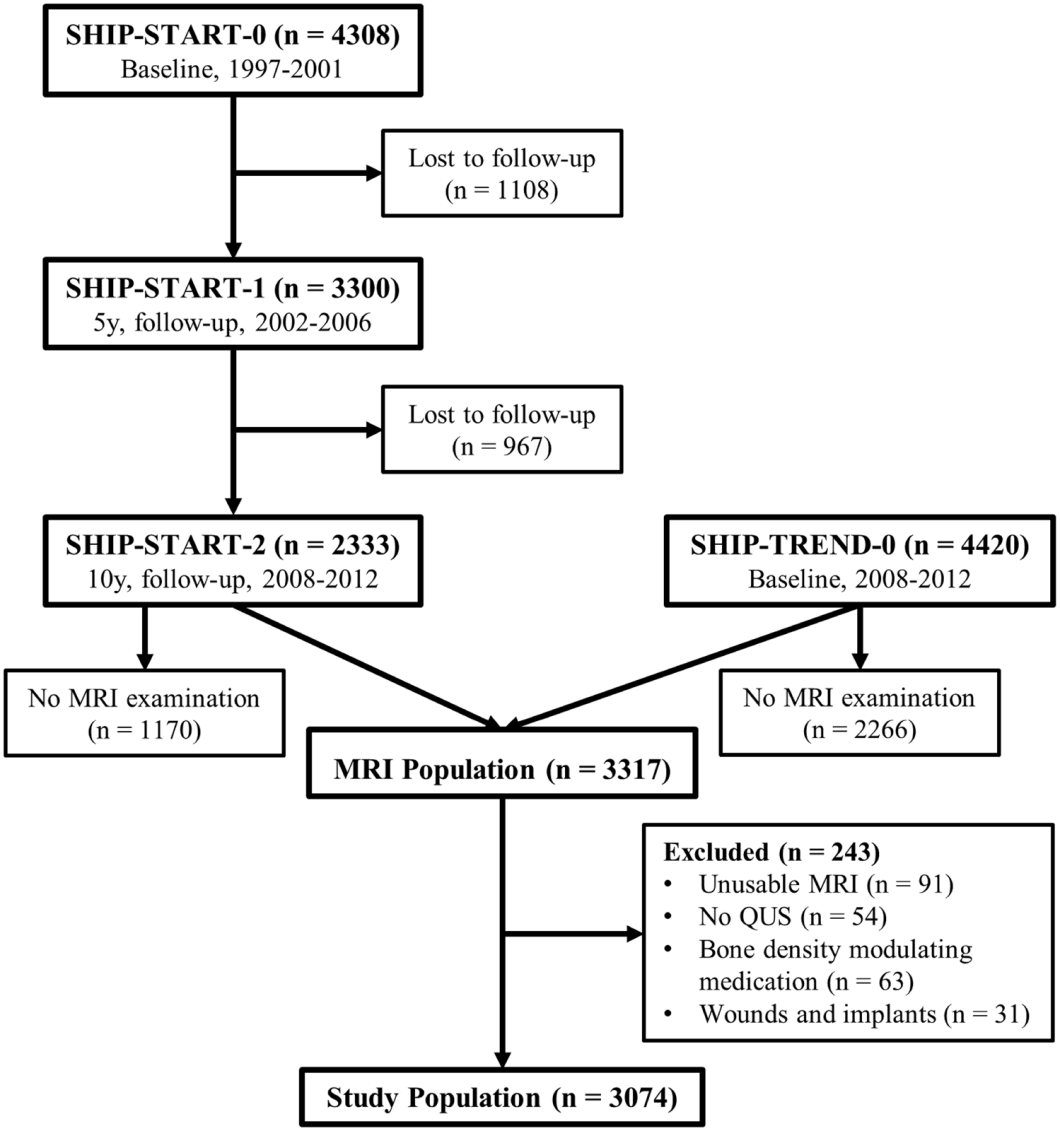
The flow diagram illustrates the selection of the study population

Quantitative ultrasound (QUS) was offered to all participants who were examined in the main examination center (Greifswald). Exclusion criteria for the present study were metal implants, prostheses below the knee including knee endoprosthesis, amputations below the knee, participants with open wounds or infections below the knee and wheelchair-bound participants. Additionally, 63 participants were excluded due to medication with bisphosphonates, selective estrogen receptor modulators, parathyroid hormone or systemic glucocorticoids. Overall, 3074 participants were eligible for the current study (Fig. 1).

### MRI protocol and angular measurements

As part of the standardized whole-body MRI, pelvic MRI was performed in a 1.5-Tesla MR scanner (Magnetom Avanto; Siemens Medical Systems, Erlangen, Germany). All MRI examinations were performed in a supine position in a standardized manner. All measurements on MRI were performed by CF, blinded to all information about the participants, using OsiriX version 5.8.5 (PIXMEO; Bernex, Switzerland). The CE, NSA and alpha angle were measured on a coronal planar image through the center of the femoral head, which was identified by using axial slices simultaneously. Detailed information on the measurement techniques and the sequence specifics were published previously [27, 28].

### Quantitative ultrasound

Ultrasound measurements were performed on both calcanei using a bone ultrasonometer (Achilles InSight, GE Medical Systems Ultrasound; GE Health Care, Chalfont St Giles, United Kingdom). Two devices without systematic differences were used. Alcohol was used as the coupling agent. The broadband ultrasound attenuation (BUA), which is the frequency-dependent attenuation of the sound waves, and the speed of sound (SOS) were measured. The stiffness-index was calculated as (0.67 × BUA + 0.28 × SOS) − 420. It describes the individual stiffness, which is then compared to the reference mean stiffness of young adults. This approach is analogue to the concept of the T-score value estimated from dual-energy X-ray absorptiometry (DXA). The reference stiffness was provided by Achilles InSight and contains sex-specific data for Germany. Based on the stiffness-index, the risk of osteoporotic fractures was divided in three categories: high risk (below reference mean minus 2.5 SDs), moderate risk (reference mean minus 1 to 2.5 SDs) and low risk (higher than reference mean minus 1 SD). For all statistical analyses, data from the foot with the lower stiffness-index was used. All measurements were performed by trained examiners on both feet on seated participants. All examiners were certified and underwent annual recertifications.

### Statistics

For reliability assessment of the hip geometric parameters, 25 cases were measured twice for intraobserver variability (CF) and a third time (JL) for interobserver variability. Variability was calculated by Bland and Altman plots with results between − 0.49 ± 2.46% and 1.78 ± 4.35% (mean of difference ± SD), showing that a good quality standard was achieved.

For the QUS, each examiner performed two measurements on the right foot of five volunteers for the annual recertification. Intraobserver variability was determined with BUA 2.98%, SOS 0.39%, and stiffness-index 2.74%. The interobserver variability was BUA 3.47%, SOS 0.36%, and stiffness-index 3.29%.

Descriptive statistics such as median and percentiles were used to describe the cohort, stratified by QUS-based osteoporotic fracture risk and sex. Associations between the QUS-based and the hip geometric parameters were assessed by multivariable linear regression models. In the regression models, BUA, SOS and stiffness-index were used as standardized variables to make the regression coefficients comparable. Various associations between the hip geometric parameters and sex, age, body height, body weight and BMI were previously detected and reported [27, 28]. Consequently, an adjustment to those factors was performed. Fractional polynomials (FP) were tested to detect potential non-linear associations. The dose–response relation was found using FP up to degree 2 with all possible combinations of powers selected from the set (− 2, − 1, − 0.5, 0, 0.5, 1, 2, 3) and were compared using the log likelihood to determine the best-fitting model. If none of the FP models fitted the data significantly better than the linear model, linear regression was applied. In the present analyses no non-linear associations became apparent. Main effects with a p < 0.05 were considered as statistically significant. Interactions between the explanatory variables and age or sex were tested for each outcome and p-values < 0.1 were considered as statistically significant. Significant interactions with age were plotted as β-coefficients estimated directly from the interaction model using the margins command. All statistical analyses were performed using Stata 17.0 (Stata Corp., College Station, TX, USA).

## Results

The median age of the 3074 participants (50.8% females) was 52 years (25th percentile: 42 years; 75th percentile: 63 years). In males, higher medians of body height, body weight and BMI than in females were observed (data not shown). 2021 participants had a low fracture risk (48.7% females). The moderate risk group consisted of 912 participants (53.5% females), while 141 participants had a high fracture risk (63.1% females).

The center-edge angle’s median of the whole sample was 30.99° with increasing medians from patients with low to high QUS-based osteoporotic fracture risk, while the neck-shaft angle’s median was 127.0° with decreasing medians from the low- to the high-risk group. The median alpha angle was 53.0° with no relevant differences between the risk groups. The complete descriptive results divided by sex and fracture risk are shown in Table 1. With higher estimated QUS-based fracture risk, the proportion of females increased. Among individuals with low, moderate and high risk, 48.7%, 53.5% and 63.1% were female, respectively. Regarding the QUS parameters, men had higher BUA (117 vs. 108 dB/MHz, p < 0.05) and stiffness-index values (96 vs. 90, p < 0.05) than women.

**Table 1:** Characteristics of the study population stratified by sex and QUS-based osteoporotic fracture risk

	Males			Females		
	Low risk	Moderate risk	High risk	Low risk	Moderate risk	High risk
N	1037	424	52	984	488	89
Age [years]	51 (41; 63)	54 (44; 66)	60 (52; 71)	48 (40; 58)	58 (48; 66)	64 (59; 71)
Height [cm]	177 (172; 180)	177 (172; 181)	176 (172; 181)	165 (160; 169)	163 (158; 167)	162 (156; 166)
Weight [kg]	87 (79; 97)	85 (77; 95)	83 (75; 95)	71 (64; 82)	71 (63; 80)	65 (60; 75)
BMI [kg/m^2^]	27.9 (25.6; 30.7)	27.4 (25.2; 29.8)	26.7 (24.4; 30.5)	26.4 (23.4; 30.5)	26.6 (23.5; 30.2)	25.7 (22.6; 29.2)
Center-edge Angle [°]	29.3 (24.9; 33.8)	31.7 (27.3; 35.9)	36.0 (30.1; 41.0)	30.6 (25.7; 35.0)	32.9 (28.7; 37.7)	34.7 (31.6; 38.7)
Neck shaft Angle [°]	127 (122; 132)	126 (122; 131)	125 (120; 129)	128 (124; 132)	126 (122; 131)	125 (121; 130)
Alpha Angle [°]	55.3 (50.7; 62.5)	57.5 (52.5; 63.2)	56.8 (51.6; 61.3)	50.7 (47.2; 54.9)	50.6 (47.4; 54.7)	50.8 (47.8; 55.0)
BUA [dB/MHz]	123 (116; 131)	105 (101; 109)	93 (89; 98)	116 (109; 124)	99 (94; 103)	86 (82; 91)
SOS [m/s]	1576 (1561; 1599)	1533 (1522; 1543)	1501 (1493; 1510)	1576 (1562; 1597)	1539 (1529; 1548)	1509 (1501; 1516)
Stiffness-index	103 (95; 115)	80 (75; 84)	63 (59; 67)	98 (91; 110)	78 (73; 82)	62 (58; 64)

Data are expressed as median, 25th and 75th percentile

**Fig. 2:**
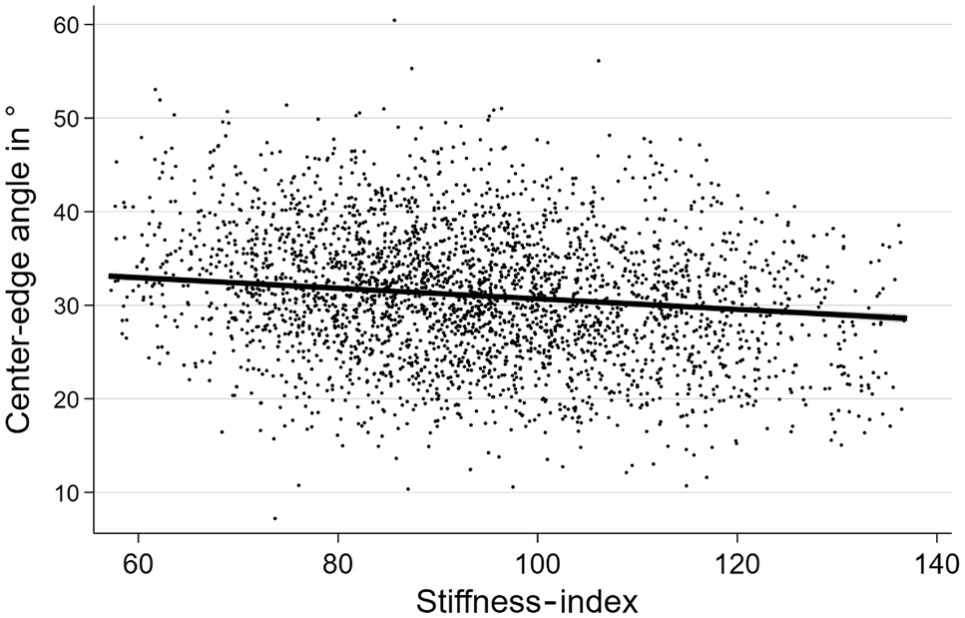
The association of the stiffness-index with the center-edge angle is shown. The black line illustrates the regression slope from the adjusted linear regression model

**Fig. 3:**
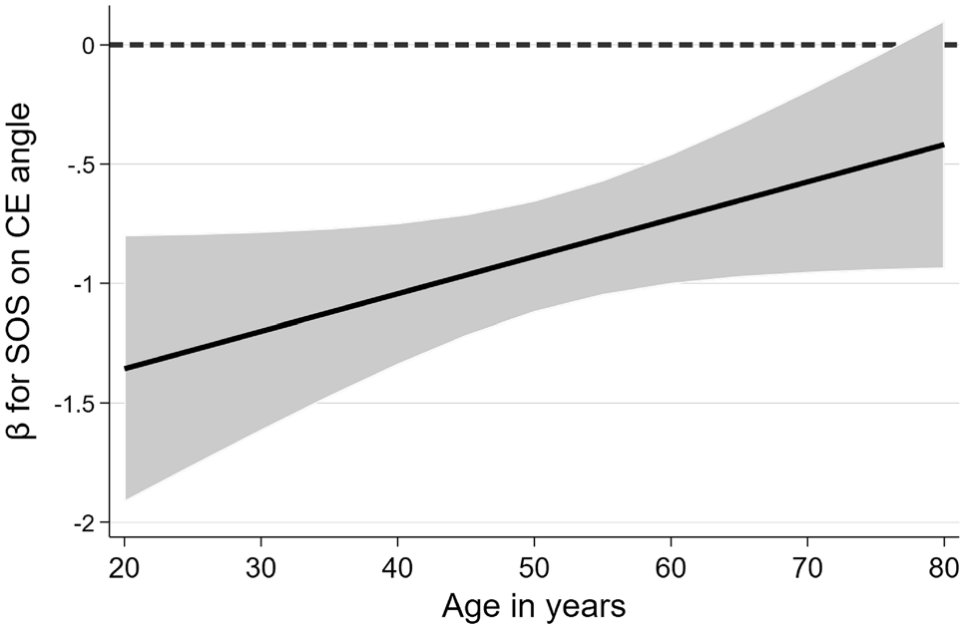
The effect modification of age on the association between standardized SOS and center-edge angle is shown. The black line illustrates the β-coefficient from the adjusted linear regression model. The grey shaded area illustrates the 95% confidence interval. The black dashed line is a reference line indicating a null effect. *CE* center-edge, *SOS *speed of sound

In fully adjusted linear regression models, inverse associations between BUA, SOS as well as stiffness-index and the CE were detected (Table 2). Higher bone stiffness was thus related to lower CE values (Fig. 2). Accordingly, individuals with moderate or high QUS-based fracture risk had higher estimated CEs than individuals with low QUS-based fracture risk (Table 2). In further analyses, a significant interaction between sex and BUA was detected in the model for CE (p = 0.028). In men, the effect of BUA on CE was stronger than in women (Men: β = − 1.25; 95% confidence interval [CI] = − 1.59 to − 0.91; p < 0.001; Women: β = − 0.73; 95%-CI = − 1.06 to − 0.40; p < 0.001). Another interaction was found between age and SOS. As demonstrated in Fig. 3, the inverse effect of SOS on center-edge angle is stronger in younger participants and turns insignificant in subjects aged 75 years or older.

**Table 2:** Associations of QUS-based parameters with hip geometric parameters

	Alpha angle	CE angle	NSA
BUA	− 0.09 (− 0.38; 0.20); p = 0.546	− 0.98 (− 1.22; − 0.74); p < 0.001	0.18 (− 0.06; 0.43); p = 0.137
SOS	− 0.16 (− 0.44; 0.12); p = 0.274	− 0.87 (− 1.10; − 0.63); p < 0.001	0.21 (− 0.03; 0.45); p = 0.081
Stiffness-index	− 0.14 (− 0.42; 0.15); p = 0.344	− 1.01 (− 1.25; − 0.78); p < 0.001	0.22 (− 0.02; 0.46); p = 0.074
Risk classification			
Moderate vs. low	0.43 (− 0.18; 1.04); p = 0.163	1.28 (0.78; 1.79); p < 0.001	− 0.60 (− 1.10; − 0.09); p = 0.022
High vs. low	0.38 (− 0.96; 1.71); p = 0.581	2.54 (1.43; 3.65); p < 0.001	− 0.52 (− 1.63; 0.60); p = 0.365

Data are expressed as β-coefficients for one standard-deviation difference in the exposure variable (95% confidence interval), derived from linear regression models adjusted for age, sex, body height and body weight

For the NSA, an insignificant positive trend with BUA, SOS and stiffness-index was present. Accordingly, individuals with moderate or high QUS-based fracture risk had lower estimated neck-shaft angles than individuals with low risk, but only the association with moderate risk was statistically significant. Finally, none of the QUS-based parameters was associated with alpha angle (Table 2).

## Discussion

Patients with reduced BMD and altered hip geometry are vulnerable for hip pathologies. Information on associations between bone quality and hip geometric parameters might be valuable for the identification of patients at risk for hip pathologies. The present study investigated associations between hip geometry and QUS-based bone quality in a large sample of the population-based SHIP study. A significant inverse association between QUS-based BUA, SOS or stiffness-index and center-edge angle, as well as a corresponding positive association of QUS-based osteoporotic fracture risk and center-edge angle was detected. Moreover, a trend towards an inverse association between the QUS-based osteoporotic fracture risk and neck-shaft angle was revealed, while associations with alpha angle were absent.

Hip dysplasia leading to osteoarthritis and proximal femur fractures are a major socioeconomic problem. Several studies suggested a greater BMD in patients with osteoarthritis of the hip [12, 13] while others did not discover any association [29]. Regarding the direct association between BMD and hip dysplasia, few and opposing results are described. Okano et al. observed higher BMDs for dysplastic hips on DXA of different skeletal sites by investigating 40 women with hip dysplasia and 31 healthy women [30]. Irie et al. measured the subchondral BMD in dysplastic hips on CT-osteoabsorptiometry. No differences were revealed between the percentage of high-density areas in the borderline dysplastic-, mild dysplastic- and control-group [31]. In contrast, Obermayer-Pietsch et al. discovered significantly lower BMD on hip DXA for dysplastic hips among 240 women, whereof 31 had a history of conservatively treated dysplasia and four underwent surgery. Their data suggested that congenital hip dysplasia is associated with a 6.3-fold increased risk for low BMD at the hip [32]. The present data showed a significant inverse association between QUS and CE. This means less dysplastic hips had a lower stiffness-index which supports the results of Okano et al. [30]. An explanation for this observation might be the increased rate of osteoarthritis in dysplastic hips [8], as osteoarthritis is associated with higher BMD [12, 13]. Interestingly, in the present study, the effect of BUA on the CE was stronger for men than women, while the effect of SOS on the CE decreases with age. No obvious explanation is present for this interaction, so further studies might be needed. In patients with subchondral insufficiency fractures of the femoral head, Ishihara et al. discovered lower CEs and therefore a higher degree of hip dysplasia [33]. Iwasaki et al. expanded these results by assessing the BMD, represented by the T-score, and the CE as significant prognostic factors for subchondral insufficiency fractures [14]. The QUS-based osteoporotic fracture risk of the present sample showed significantly higher CE in higher fracture risk groups.

Low BMD is generally accepted as an important risk factor for femoral neck, as well as trochanteric fractures [15, 16]. The prospective study of Fox et al. revealed an increase of fracture risk by 49% per standard deviation decrease of BMD [34]. Additionally, there is more and more evidence that specific proximal femur geometry increases fracture risk as well. Thalmann et al. revealed that the NSA influences fracture site and type [35], while Gnudi et al. determined age, NSA and BMD as independent predictors for hip fractures. They proposed site-matched BMD and NSA as the best predictors for proximal femur fractures in postmenopausal females [36]. For males, the association between NSA and hip fractures was determined as well, but it was not independent of BMD [37]. In the present sample, an inverse association of moderate vs. low fracture risk and NSA was revealed. Thus, individuals with moderate QUS-based fracture risk had lower NSA than those with low fracture risk. Yet, the association of high vs. low fracture risk and NSA missed statistical significance. This lack of significance between the low and high fracture risk group might be explained by the different group sizes, i.e., n = 2021 with low and n = 141 with high fracture risk. Previous studies described opposing results. Some authors described lower BMD [17, 38] and higher NSA in fracture cases [17, 38, 39] and the association to NSA was explained by the longer lever arm during the lateral impact [17], while others described narrower NSA in the fracture group [19]. However, knowledge on associations between QUS-based fracture risk and NSA might be beneficial since the combination of BMD and geometric parameters seem to improve the risk prediction for proximal femur fractures [17–19, 36]. Evidence for associations between bone properties and NSA are, however, controversial. Thus, a lack of strong correlation between BMD and NSA was reported from other studies [18, 19, 38]. Kroge et al. found reduced bony microarchitecture, mineralization and osteocyte properties, independent of the NSA on 28 osteoarthritic hips undergoing total hip arthroplasty [40]. In contrast, Kobayashi et al. revealed an association of the NSA and a decreased BMD in the distal subtrochanteric bone on osteoarthritic hips [41] while Zhang et al. [42], Ripamonti et al. [37] and Machado et al. [43] discovered an inverse correlation between NSA and BMD. On the other hand, Masuhara et al. described a positive correlation of the NSA with the BMD [44]. For hip fractures, NSA and BMD are sometimes described as independent risk factors [36], sometimes as dependent risk factors [37]. Since opposing results are already published, it is not surprising that the present sample showed no significance, but only a positive trend between BUA, SOS or stiffness-index and NSA. However, knowledge on the associations and interactions between bone quality, assessed by QUS, and hip geometry may be useful for prevention.

Cam-Type deformity and following FAI might lead to early osteoarthritis of the hip [6]. Since there is a positive association between an extreme elevation in BMD and osteoarthritis [45], data about associations between Cam-Type deformity and bone quality are needed. So far, only few studies were performed on this topic. Speirs et al. discovered an increased acetabular subchondral bone density in patients with Cam-type deformities. Interestingly, the severity of the deformity had a higher correlation with the BMD than the symptom status of the patients [46]. The same workgroup detected a positive association between BMD and Cam-type deformities for the subchondral bone of the femoral head [47]. With 12 participants, the sample size for each group was relatively small. In contrast, Zucker et al. showed that high bone mass and Cam morphology are independently related to hip osteoarthritis by investigating 694 hips, of which 20,6% had a Cam deformity [48]. Regarding the direct association between BMD and alpha angle, Beaulé et al. detected a positive correlation for the antero-superior region of the acetabulum. These results were obtained on a small sample (36 participants) as well [49]. The present study detected no significant association between the QUS and the alpha angle.

The present study has several limitations. The population investigated is a subsample of the SHIP-START-2 and SHIP-TREND-0 cohort. MRI participants differed from non-MRI participants considerably in terms of sociodemographic characteristics but differences regarding health utilization were small [50]. Since participants with implants on the lower extremities, including the hip, were excluded, the participants might represent a healthier sample than the normal population. Therefore, the results might not apply to a more morbid population. Moreover, the low rate of hip pathologies observed among the study population, might have prevented the detection of further associations. A second limitation is the assessment of the hip geometry on non-rotation corrected, two dimensional coronal MRI. As previously discussed, the obtained values are in accordance with other acquisition techniques [27, 28]. Therefore, the hip geometric parameters can be considered reliable. The cross-sectional study design limits our findings to associations but no cause-and-effect relationships.

A further limitation is the use of QUS for the assessment of the bone quality. DXA is the gold standard in diagnostics of osteoporosis, but the radiation exposure was not justified for participants in a population-based project. However, calcaneal QUS correlates strongly with whole body DXA in adults [51] and with calcaneal DXA measurements [52]. Li et al. demonstrated that calcaneal QUS has a high value in osteoporosis-screening. In their study on femoral DXA and calcaneal QUS, the sensitivity and specificity for the diagnosis of osteoporosis was 100% when calcaneal QUS T-score was ≤ –2.35 [24]. Moreover, QUS allows estimations on fracture risk due to osteoporosis [24, 51, 53]. Khaw et al. showed that QUS of the calcaneus predicts total fracture risk and hip fracture risk in a continuous relation [23]. BUA might additionally reflect structural features of the bone [22]. Especially when combined with clinical risk factors, an estimation of the probability of osteoporotic fractures is possible [54]. Another limitation might be that ultrasound measurements are observer dependent. However, the QUS were assessed on the calcaneus which has few soft tissues and a large parallel plane for easy measurement. Moreover, all calculations are made by the ultrasound device and good reliability and validity is described [55]. Therefore, QUS are considered advantageous because of no-dose radiation, portability, and the simple operation [24]. Therefore, the combination of altered geometry of the hip and bone quality assessed by quantitative ultrasound could be advantageous for assessing the estimated fracture risk more precisely without radiation. There might be no benefit for already fractured hips. However, calcaneal QUS has good specificity as a screening tool for osteoporosis and can be recommended, especially to reduce the number of DXA [24]. With additional information on hip geometry and its associations, vulnerable patients might be assessed more efficiently without any radiation. For these patients, further diagnostics and adequate therapy for osteoporosis and fall prevention can be established.

To conclude, several associations between QUS-based bone properties, including osteoporotic fracture risk and hip geometry are present. The inverse association between QUS parameters and center-edge angle showed that more dysplastic hips had increased QUS values. This might be explained by the increased rate of osteoarthritis in dysplastic hips while osteoarthritis is associated with higher BMD. Additionally, an increasing QUS-based fracture risk was positively associated to the CE, while more valgus hips had a lower fracture risk. For the alpha angle no significant association with any of the QUS parameters was present. The knowledge on the reported associations and interactions may be useful for prevention, so vulnerable groups might be detected without the exposure to further radiation if MRI is present and suitable therapy can be considered. For evidence on cause-effect relationships, especially on fractures, longitudinal studies are needed.

## Data Availability

The data that support the findings of this study are not openly available due to reasons of sensitivity and are available from the corresponding author upon reasonable request. Data are located in controlled access data storage at the Institute of Community Medicine Greifswald.
